# The Impact of Fecal Diversion on Immune Checkpoint Inhibitor Adverse Gastrointestinal Toxicities †

**DOI:** 10.3390/jcm14134711

**Published:** 2025-07-03

**Authors:** Saltenat Moghaddam Adames, Sidra Naz, Jianliang Dai, Yinghong Wang, Anusha Shirwaikar Thomas

**Affiliations:** 1 Department of Internal Medicine, Baylor College of Medicine, Houston, TX 77030, USA; smoghaddam@mdanderson.org; 2 Department of Gastroenterology, Hepatology, and Nutrition, The University of Texas MD Anderson Cancer Center, Houston, TX 77030, USA; snaz@mdanderson.org (S.N.); ywang59@mdanderson.org (Y.W.); 3 Department of Biostatistics, The University of Texas MD Anderson Cancer Center, Houston, TX 77030, USA; jdai4@mdanderson.org

**Keywords:** immune checkpoint inhibitors, colorectal cancer, immune-related adverse event, colitis

## Abstract

**Background/Objective:** Immune checkpoint inhibitors (ICIs) are highly effective cancer therapies used across a broad spectrum of malignancies. They function by disrupting immune inhibitory pathways, resulting in an amplified immune response against tumors. However, this heightened immune activity can predispose patients to immune-mediated colitis (IMC), which is graded using the Common Terminology Criteria for Adverse Events (CTCAE) and can range from mild diarrhea to severe colitis. Prior studies have shown that fecal stream diversion can modify the gut microbiome and influence the severity of intestinal inflammation. This study investigates the impact of fecal stream diversion on IMC outcomes in cancer patients receiving ICIs. **Methods:** We conducted a retrospective cohort study of patients treated with ICIs from 2016 to 2023 who had a history of fecal stream diversion. Demographic, oncologic, and toxicity-related data were collected. Patients with active gastrointestinal infections, autoimmune GI diseases, or graft-versus-host disease were excluded. Descriptive statistics and univariate and multivariate logistic regression analyses were performed using SAS version 9.4. **Results:** A total of 44 patients were included and categorized into two groups based on the timing of bowel stoma creation relative to the IMC event. CTCAE grade for diarrhea was used to assess GI toxicity. While overall CTCAE grade distribution for diarrhea did not differ significantly between groups (*p* = 0.22), Hispanic ethnicity was significantly associated with a lower CTCAE grade compared to non-Hispanic or Latino individuals (OR [95% CI] = 0.12 [0.02, 0.62], *p* = 0.011). In contrast, higher CTCAE grades were significantly associated with ileostomy versus colostomy (OR [95% CI] = 3.21 [1.01, 10.18], *p* = 0.048) and in patients without an ostomy at the time of diarrhea onset compared to those with an ostomy (OR [95% CI] = 8.87 [2.51, 31.31], *p* = 0.0007). **Conclusions:** Our findings suggest that the CTCAE diarrhea grade is significantly associated with ethnicity, type of stoma, and presence of ostomy at the time of diarrhea. Limitations include the retrospective study design and small sample size. These results contribute to understanding potential strategies for mitigating the serious gastrointestinal toxicities of ICIs.

## 1. Introduction

Immune checkpoint inhibitors (ICIs), such as anti-PD-1/PDL-1 and anti-CTLA-4, are highly efficacious cancer therapies used to treat a wide variety of malignancies. These agents are a significant breakthrough in the field of cancer immunotherapy [1]. ICIs work by blocking immune checkpoints and triggering an unmitigated immune response to target cancer cells [2]. However, this excessive immune activation can predispose patients to immune-related adverse events (irAEs) [3]. The gastrointestinal (GI) tract is frequently involved in immune checkpoint inhibitors’ toxicity and can manifest as immune checkpoint-inhibitor-mediated colitis (IMC). Although any portion of the GI tract can be affected, toxicity is more commonly observed in the colon [4]. The severity of ICI toxicity on the GI tract can vary from mild diarrhea to a more aggressive colitis. This toxicity is graded using the Common Terminology Criteria for Adverse Events (CTCAE) classification system. Severe IMC often interrupts cancer therapy, increases morbidity and mortality, and reduces quality of life [5].

Best practice advice suggests IMC is often effectively managed with immunosuppression, including systemic corticosteroids and biologic therapy such as vedolizumab and infliximab [6]. However, it is not uncommon to see IMC refractory to standard therapies such as steroids and biologics. Emerging data show fecal microbiota transplantation (FMT) is promising for IMC treatment, highlighting the gut microbiome’s role in ICI efficacy and colitis severity [7]. FMT has paved the way for more inquiry into the relationship between the gut microbiome and IMC.

Studies show that surgical changes associated with diversion of the fecal stream can positively alter the gut and colon microbiome. Surgical diversion, such as loop ileostomy, is effective for acute inflammatory colitis and may serve as an alternative to emergent colectomy [8]. A case report published in the Journal of Immunotherapy has shown a favorable response and complete recovery in treating IMC with a diverting loop ileostomy. The development of a healthy gut microbiome with an ostomy is often seen in patients with inflammatory bowel disease (IBD). Ostomy surgery in IBD patients reduces mucosal inflammation and helps restore microbial balance [9]. This stable post-ostomy environment is shown to foster a less pathogenic microbial community [10]. This microbiota shift has been shown to lower the risk of recurrent inflammation [11]. In IBD, an ostomy may support mucosal healing and disease control by improving gut microbiome health. As such, diversion ostomy might be a valid treatment option in severe or recurrent colitis due to ICI therapy [12].

Few studies have investigated the association between the alteration of the gut microbiome via diversion bowel stomas and IMC severity. In this retrospective cohort study, we explored the association between the severity of IMC in cancer patients with diversion of the fecal stream.

## 2. Materials and Methods

### 2.1. Study Design and Population

This is a descriptive, single-center, retrospective cohort study that includes patients diagnosed with cancer from 2016 to 2023 at a tertiary cancer center. This study was approved by the institutional review board; protocol PA18-0472 (20 February 2018). Consent was waived due to the retrospective nature of the analysis. We identified cancer patients 18 years or older who (1) were treated with ICIs for cancer, (2) were diagnosed with immune-mediated colitis, and (3) have a history of diversion ostomy. Data on demographic, oncologic and toxicity variables were collected. Patients with pre-existing or active inflammatory bowel disease (IBD), GI infection and those with graft versus host disease were excluded.

### 2.2. Clinical Data

Demographic data, including age, sex, race and ethnicity, were collected. Data on cancer type, ICI type (anti-CTLA-4 monotherapy, anti-PD-1/L1 or combined therapy) and stoma type (ileostomy, colostomy or both) were collected. Chronological data on the presence of stoma before and after the IMC event were collected. Common Terminology Criteria for Adverse Events (CTCAE) peak symptoms for diarrhea were calculated for all patients. CTCAE grade 1 for diarrhea is defined as increase of <4 stools per day and mild increase in ostomy output compared to baseline, grade 2 for diarrhea is defined an increase of 4–6 stools per day and moderate increase in ostomy output compared to baseline; limiting activities of daily living, grade 3 diarrhea is an increase of ≥7 stools per day over baseline, severe ostomy output and hospitalization, grade 4 is defined as life-threatening consequences; urgent intervention indicated and grade 5 diarrhea is defined as diarrhea leading to death [13]. Diagnosis of ICI colitis was based on the patients’ clinical presentation and history, exclusion of other diagnoses, and in some cases, histological features on endoscopic evaluation.

### 2.3. Statistical Analysis

The statistical analyses performed were descriptive in nature. Categorical variables were summarized by count and percentage and continuous variables were described by median and interquartile range (Q1, Q3). Statistical associations between outcome and predictors were analyzed by univariate and multivariate logistic regression models. The significance level used in this analysis was *p* < 0.05. Statistical analyses were conducted using SAS (Statistical Analysis System version 9.4 for Windows Copyright ©2016 by SAS Institute Inc., Cary, NC, USA).

## 3. Results

### 3.1. Patient Population, Characteristics and Oncologic History

**Figure 1:**
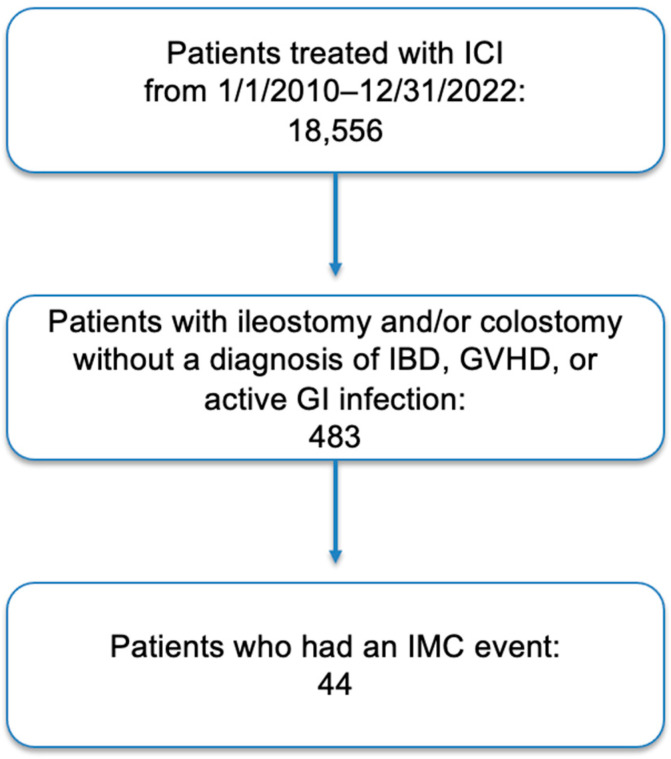
Summary of the selection process for patients included in the study. Abbreviations: ICI; immune checkpoint inhibitor, IBD; irritable bowel disease, GVHD; graft-vs-host-disease, GI; gastrointestinal, IMC; immune-mediated colitis.

Out of 483 patients, 44 patients were identified who met our inclusion and exclusion criteria (Figure 1). Patients had a median age of 59 years (+/− SD) with 25 (56.8%) patients being male and 37 (84.1%) patients being white. Four (9%) patients identified as Hispanic or Latino. As for oncologic history, 11 (25%) patients had melanoma, 11 (25%) patients had a genitourinary cancer, 1 (2.3%) patient had lung cancer, 13 (29.5%) patients had gastrointestinal cancer, 2 (4.5%) patients had endocrine cancer, 1 (2.3%) patient had a hematological malignancy, 3 (6.8%) patients had a gynecologic malignancy, and 2 (4.5%) patients had another type of cancer. Of the patients with gastrointestinal or genitourinary malignancies, 5 (20.8%) received abdominal or pelvic radiotherapy prior to or during ICI treatment (Table 1).

### 3.2. Characteristics and Treatment of Colitis

These 44 patients were categorized into 2 subgroups based on the timing of the stoma and IMC event. As for ICI type, 3 (6.8%) patients were treated with anti–CTLA-4 monotherapy, 21 (47.7%) patients were treated with anti–PD-1/L1 monotherapy and 20 (45.5%) patients were treated with combination anti–CTLA-4 and anti–PD-1/L1. As for ostomy data, 12 (27.3%) had ileostomies, 25 (56.8%) had colostomies, and 7 (15.9%) had both ileostomy and colostomies. Patients had stomas in the setting of primary GI or metastatic cancer resection, bowel obstruction, palliation of metastatic disease or unspecified reasons. As for our treatment groups, group 1 had a stoma placed before an immune-mediated colitis event, 28 (63.6%) patients. In this group, 6 (21.4%) patients had an ileostomy, 18 (64.2%) had a colostomy and 4 (14.2%) had both. Group 2, 16 (36.4%) patients had a stoma placed after the immune-mediated colitis event. In this group, 6 (37.5%) patients had an ileostomy, 7 (43.8%) had a colostomy and 3 (18.8%) had both (Table 1). This study did not have a control group.

Of our sample, the median time of duration from ICI exposure to IMC event was 234 (123–322) days. In total, 26 (59.1%) patients required steroids with a median of 3.8 (2.9–4.9) tapering events. Of these 26 patients, 9 (36%) had ileostomies, 12 (46.2%) had colostomies and 5 (20%) had both. In total, 14 (31.8%) patients required biologics with 7 (15.9%) receiving at least 2 doses; of these 14 patients, 3 (21.4%) had ileostomies, 8 (57%) had colostomies and 3 (21.4%) had both. Only 2 (4.5%) patients received FMT for IMC treatment; one had both colostomy and ileostomy, and one had a colostomy. The median fecal calprotectin value available on 20 patients at the time of colitis was 493 mcg/g (385–603). Approximately 30 (68.2%) patients resumed ICI for cancer treatment and 21 (47.7%) had a recurrence of an IMC event. In total, 11 (25%) patients in our sample had a history of non-steroidal anti-inflammatory drugs or proton pump inhibitor use. At the conclusion of our analysis, 9 (20.5%) patients had stable disease, 2 (4.5%) patients had no evidence of disease, 7 (15.9%) had progression of disease, and 24 (54.5%) had expired (Table 2).

### 3.3. Treatment of Colitis per Group

For IMC event treatment, group 1 had 12 (42.9%) patients treated with steroids, 11 (39.3%) patients treated with biologics and 1 (3.6%) patient treated with FMT. Group 2 had 14 (87.5%) patients treated with steroids, 3 (18.8%) patients treated with biologics and 1 (6.3%) with FMT. Patients in group 1 experienced fewer refractory episodes compared to patients in group 2, 12 (42.9) vs. 9 (56.3). However, fewer patients required treatment with biologics in group 2 (Table 3). The therapeutic choice for each patient was determined individually by the treating physician based on clinical judgment.

We analyzed the CTCAE grade for diarrhea as an indicator of GI toxicity. The average CTCAE grade of diarrhea in those with a stoma placed before the IMC event was 2.07 and stoma placed after the IMC event was 2.63. Although there is a lower CTCAE grade of diarrhea, the results indicate that the distributions of CTCAE grade for diarrhea (*p* = 0.22) were not significantly different amongst the 2 cohorts. However, Hispanic and Latino ethnicity was associated with a significantly lower grade of diarrhea (OR (95% CI) = 0.12 (0.02, 0.62), *p* = 0.011). Compared to the presence of a colostomy, an ileostomy was significantly associated with a higher CTCAE grade of diarrhea. A higher CTCAE grade of diarrhea was significantly associated with the absence of ostomy at the time of toxicity. (OR (95% CI) = 8.87 (2.51, 31.31), *p* = 0.0007) (Table 4).

### 3.4. Endoscopic Features

Of the 44 patients, 21 had endoscopic assessment; 7 (33.3%) patients had ulcerative findings, 8 (38.1%) had non-ulcerative findings and 5 (23.8%) had the appearance of normal mucosa. Of the 17 histology samples available, 8 (47.1%) patients had evidence of acute inflammation, 7 (41.2%) had chronic inflammation and 2 (11.8%) had lymphocytic changes (Table 5). Ileostomies were commonly associated with ulcerative and acute inflammatory changes on endoscopy, and colostomies were associated with non-ulcerative, normal mucosa and chronic inflammatory changes on endoscopy.

## 4. Discussion

Colitis is an inflammatory disorder of the bowel secondary to infections, allergic reactions, ischemia, autoimmune disorders such as Crohn’s and ulcerative colitis, medications and radiation [14]. Colitis varies in severity and untreated colitis can lead to life-threatening complications such as bowel perforation and sepsis [15]. Immune checkpoint inhibitors have revolutionized the landscape of management of a variety of malignancies, yet predispose to several immune-related adverse events. IMC is the most frequently encountered such toxicity, which can gravely affect the quality of life of these immunocompromised cancer patients [16].

The overall rate of IMC in patients treated with ICI for any cancer, particularly grade 3–4, is well above 10% [17]. Systemic corticosteroids and biologic therapy have proven to be efficacious; however, many patients are refractory to standard therapies [18]. In a case series following 13 patients with ICI colitis, 33% percent of patients required surgical intervention resulting in an end ileostomy despite treatment with infliximab and achieved improvement of colitis [19]. Understanding this mechanism lies in understanding the gut microbiome’s influence on colitis.

The gut microbiome plays an important role in colitis by its involvement in the de novo synthesis of lipids that function as signaling molecules, thereby influencing local tissue metabolism. These microbial-derived lipids influence the progression of inflammation, autoimmune diseases, and other extra-GI manifestations [20]. The gut microbiota is also a predictive biomarker in ICI therapy [21]. Studies show that abnormal gut microbiota can lead to primary resistance to ICIs. There is a positive correlation between clinical responses to ICIs and the abundance of *Akkermansia muciniphila*. Supplementation of this organism, through methods such as FMT, has been shown to restore the efficacy of ICI by increasing recruitment of T-lymphocytes [22,23]. FMT fosters the development of a diverse host-microbiome profile like that of a healthy donor and augments the local commensal community in disease states, thereby facilitating treatment of IMC [24].

In our study, we observed that at the time of toxicity, our cohort had a significantly higher clinical grade of diarrhea in the absence of a diverting stoma. Furthermore, patients with a stoma placed prior to the IMC event experienced fewer refractory episodes than patients who had a stoma placed after the IMC event, which leads us to speculate about the potential role of fecal diversion in altering the gut microbiome. However, the mechanism of action behind this phenomenon is still largely unknown. Can diversion of the fecal stream confer a similar response to that of FMT?

To further evaluate this question, we look to the IBD population. Diversion of the fecal stream is commonly seen in the IBD population. It is well known that fecal diversion is associated with acute clinical remission in many patients with refractory Crohn’s colitis and severe perianal disease. In a systematic review and meta-analysis of 33 cohort studies of patients who had temporary fecal diversion for refractory Crohn’s disease, diversion resulted in clinical improvement in 61% [25]. In another study with 44 patients undergoing fecal diversion for Crohn’s disease, 70% sustained disease remission and had a significant reduction in steroid requirements [26]. Evidence suggests that the relationship between diversion of the fecal stream and clinical remission is due to the development of a healthy gut microbiome [27]. In a study evaluating diversion of the fecal stream and the evolution of the gut microbiome, it was shown that a “healthy” colonic community gradually drifted towards an “unhealthy” colonic community following 1 and 2 months after ileostomy closure [28]. One proposed mechanism suggests that the intestinal flora changes from strict anaerobes to facultative anaerobes before and after ileostomy, respectively, thereby preventing the overgrowth of competitive pathogens that are harmful to the gut [29]. In understanding the positive relationship between ostomy and gut microbiota in the IBD population, we hypothesize that our cancer cohort may have a similar response. Additional protective factors of fecal diversion in Crohn’s include decreased glycoprotein synthesis and stable crypt cell production rate (CCPR) compared to control groups [30]. These microbiota and physiologic factors can explain the protective role the fecal stream may play in inflammatory processes, as seen in our cohort. However, more studies are needed to further understand this association.

Emerging evidence suggests that gut microbiome composition differs by race and ethnicity, potentially due to genetic, dietary, and environmental factors. These variations may influence immune responses and treatment outcomes, including susceptibility to immune-related adverse events such as colitis [31]. The role of microbiota in influencing clinical outcomes is highlighted in our cohort’s Hispanic population. There is a relationship between microbiota in Hispanics vs. non-Hispanics. This difference is mainly due to lifestyle, diet and varying disease processes amongst ethnic groups. Some studies show that a traditional Hispanic diet may promote beneficial bacteria such as Prevotella and Bifidobacterium compared to a Bacteroides-dominant profile in non-Hispanic whites [32]. It has been noted in literature that the longer a Hispanic individual lives in the US and incorporates a Western diet, they often have reduced gut diversity and loss of beneficial species [33]. Furthermore, Hispanic individuals may experience different metabolic outcomes, potentially linked to gut microbiota [34]. However, our described association between Hispanic/Latino ethnicity and lower diarrhea grade should be interpreted as exploratory, given the sample size.

Our study provides novel information that suggests diversion of the fecal stream may influence the gut microbiota, possibly enough to influence the severity of IMC. However, significant limitations exist with this small, single-centered cohort as it can limit statistical power and increase the risk of Type I error, sometimes affecting its reproducibility and generalizability in larger cohorts. Other limitations include the retrospective nature of the study, lack of microbiome data and the possible presence of diversion and radiation colitis, as it may present similarly to IMC. However, we accounted for diversion colitis by carefully evaluating the timeline of colitis compared to stoma placement and ICI initiation, and correlated it with endoscopic imaging and biopsies when possible. Moreover, the presence of colitis in patients with GI and GU malignancies who received radiotherapy introduces potential confounding from radiation-induced enterocolitis, limiting the interpretability of our observed GI toxicity findings. Furthermore, it is important to note that fecal stream diversion is a major surgical intervention, and special considerations must be made, such as the impact on quality of life and the psychosocial burden of having an ostomy. These decisions should involve shared decision-making between patients and providers, particularly in the context of balancing cancer therapy-related toxicities with the risks and long-term consequences of surgical intervention

## 5. Conclusions

This study suggests that diverting the fecal stream may reduce the severity of IMC in cancer patients receiving immune checkpoint inhibitors, possibly by altering the gut microbiota. A temporary diversion of the fecal stream may serve as a novel therapeutic option for IMC refractory to standard medical treatment. Further research is needed to explore the mechanisms behind this relationship and validate the therapeutic potential of fecal diversion in managing IMC.

## Figures and Tables

**Table 1:** Overview of the characteristics of the 44 patients included in the study.

Patient Characteristics, N = 44	
**Characteristic**	**No. (%)**
Age, years, median (IQR)	59 (31–86)
Sex, male	25 (56.8)
Race, white	37 (84.1)
Ethnicity, Hispanic/Latino	4 (9.0)
**Cancer type**	
Melanoma	11 (25)
Genitourinary	11 (25)
Lung	1 (2.3)
Gastrointestinal	13 (29.5)
Endocrine	2 (4.5)
Hematological	1 (2.3)
Gynecologic	3 (6.8)
Others	2 (4.5)
**Type of ICI**	
Anti–CTLA-4 monotherapy	3 (6.8)
Anti–PD-1/L1 monotherapy	21 (47.7)
Combination anti–CTLA-4 and anti–PD-1/L1	20 (45.5)
**Received abdominal or pelvic radiation before or during ICI treatment, N = 24**	5 (20.8)
Gastrointestinal cancer, N = 13	3 (23.1)
Genitourinary cancer, N = 11	2 (18.2)
**Stoma type**	
Ileostomy	12 (27.3)
Colostomy	25 (56.8)
Both	7 (15.9)
**Stoma and IMC**	
Stoma placed before IMC event	28 (63.6)
Ileostomy	6 (21.4)
Colostomy	18 (64.2)
Both	4 (14.2)
Stoma placed after an IMC event	16 (36.4)
Ileostomy	6 (37.5)
Colostomy	7 (43.8)
Both	3 (18.8)

Abbreviations: CTLA-4, cytotoxic T lymphocyte antigen 4; ICI, immune checkpoint inhibitor; IQR, interquartile range; PD-1/PD-L1, programmed cell death 1/programmed death ligand 1, IMC, immune-mediated colitis.

**Table 2:** Outcomes of the 44 patients who developed immune-mediated colitis after treatment with immune checkpoint inhibitors.

Patient Outcomes, N = 44			
Characteristics	Group 1	Group 2	Total
	No. (%)	No. (%)	No. (%)
Duration of ICI exposure to IMC event, in days, median (IQR)	310 (289–342)	123 (97–143)	234 (123–322)
**Patients treated with steroids after IMC event**	12	14	26 (59.1)
Ileostomy	5	4	9 (36)
Colostomy	4	8	12 (46.2)
Both	3	2	5 (20)
Number of steroid taper events, median (IQR)	2.9 (2.1–3.5)	4.7 (4.2–5.4)	3.8 (2.9–4.9)
**Patients treated with biologics after IMC event**	11	3	14 (31.8)
Ileostomy	3	0	3 (21.4)
Colostomy	6	2	8 (57)
Both	2	1	3 (21.4)
**Patients treated with FMT after IMC event**	1	1	2 (4.5)
Colostomy	1	0	1 (2.3)
Both	0	1	1 (2.3)
Fecal calprotectin at the time of colitis, N= 20, median (IQR)	378 (329–439)	598 (525–664)	493 (385–603) *
Patients who resumed ICI for cancer treatment	19	11	30 (68.2)
Patients who had a recurrence of IMC event	12	9	21 (47.7)
Patients who had a history of NSAID or PPI use	7	4	11 (25%)
**Cancer status**			
Stable disease	4	5	9 (20.5)
No evidence of disease	0	2	2 (4.5)
Progression of disease	5	2	7 (15.9)
Patients expired	11	13	24 (54.5)

Group 1: stoma prior to IMC event, Group 2: stoma post IMC event. Abbreviations: ICI; immune checkpoint inhibitor, IMC; immune-mediated colitis, FMT; fecal microbiota transplantation, NSAID; non-steroidal anti-inflammatory drug, PPI; proton pump inhibitor. * Fecal calprotectin based on a subset of 20 patients and measured in mcg/g.

**Table 3:** Treatment response of two groups of patients with immune-mediated colitis.

Patient Treatment Outcomes per Group, N = 44		
	Group 1, N = 28; N (%)	Group 2, N = 16 N (%)
Patients treated with steroids	12 (42.9)	14 (87.5)
Patients treated with biologics	11 (39.3)	3 (18.8)
Patients treated with FMT	1 (3.6)	1 (6.3)
Refractory IMC event post treatment	12 (42.9)	9 (56.3)

Group 1: stoma prior to IMC event; Group 2: stoma post IMC event. Abbreviations: IMC, immune-mediated colitis; FMT, fecal microbiota transplantation.

**Table 4:** Statistical analysis of factors associated with the severity of diarrhea (graded using CTCAE) in patients with immune-mediated colitis.

Descriptive Statistics of CTCAE Grade for Diarrhea Among Four Cohorts							
Variable	Comparison	Univariate Logistic Model			Multivariate Logistic Model		
		OR	*p*-Value	95% CI	OR	*p*-Value	95% CI
Gender	Female vs. Male	0.76	(0.35, 1.66)	0.490			
Race	Black vs. White	6.00	(0.37, 98.3)	0.209			
Race	Other vs. White	1.05	(0.34, 3.25)	0.933			
Patient Ethnicity	Hispanic or Latino vs. non-Hispanic or Latino	0.25	(0.06, 1)	0.050	0.118	(0.02, 0.62)	0.011
Type of diversion ostomy	ileostomy; ileostomy vs. colostomy	3.35	(1.3, 8.64)	0.012	3.211	(1.01, 10.18)	0.048
Type of diversion ostomy	colostomy; ileostomy vs. colostomy	1.43	(0.47, 4.31)	0.526	1.533	(0.45, 5.17)	0.491
Have ostomy at the time of ICI	no vs. yes	2.44	(1.1, 5.44)	0.029	0.670	(0.23, 1.98)	0.469
Have ostomy at the time of diarrhea colitis	no vs. yes	6.67	(2.54, 17.5)	<0.001	8.865	(2.51, 31.31)	0.0007
Presence of take down stoma	no vs. yes	0.66	(0.25, 1.73)	0.396			
cohort	After ICI vs. Before ICI	3.58	(1.15, 11.2)	0.028	3.053	(0.88, 10.57)	0.078
cohort	Before Tox vs. Before ICI	1.19	(0.42, 3.37)	0.743	1.360	(0.44, 4.20)	0.592
cohort	After Tox vs. Before ICI	4.73	(1.35, 16.6)	0.015	2.846	(0.73, 11.14)	0.133

Abbreviations: IMC, immune-mediated colitis; Tox, toxicity.

**Table 5:** Endoscopic features and inflammation types in two patient groups based on the timing of stoma placement relative to immune-mediated colitis onset.

Endoscopic Findings in Patients with Immune Mediated Colitis, N = 21			
**Group 1, N = 8**			
**Patient**	**Type of Stoma**	**Endoscopic Feature**	**Type of Inflammation**
1	Both	Non- ulcerative	Chronic
2	Ileostomy	Non- ulcerative	Acute
3	Colostomy	Non- ulcerative	Acute
4	Colostomy	Normal mucosa	Chronic
5	Colostomy	Non- ulcerative	Unavailable
6	Both	Non- ulcerative	Acute
7	Ileostomy	Non- ulcerative	Chronic
8	Ileostomy	Normal mucosa	Lymphocytic
**Group 2, N = 13**			
9	Colostomy	Ulcerative	Chronic
10	Colostomy	Ulcerative	Acute
11	Colostomy	Non- ulcerative	Chronic
12	Colostomy	Non- ulcerative	Chronic
13	Colostomy	Ulcerative	Lymphocytic
14	Ileostomy	Ulcerative	Acute
15	Colostomy	Normal mucosa	Unavailable
16	Colostomy	Normal mucosa	Unavailable
17	Both	Ulcerative	Acute
18	Ileostomy	Ulcerative	Acute
19	Colostomy	Normal mucosa	Chronic
20	Ileostomy	Ulcerative	Acute
21	Colostomy	Normal mucosa	Unavailable

Group 1: patients who had a stoma prior to immune-mediated colitis event; Group 2: patients who had a stoma after immune-mediated colitis event.

## Data Availability

Available upon request to the corresponding authors.
